# Optical Fiber Grating-Prism Fabrication by Imprint Patterning of Ionic-Liquid-Based Resist

**DOI:** 10.3390/ijms24021370

**Published:** 2023-01-10

**Authors:** Natalia Turek, Piotr Pala, Andrea Szpecht, Adrian Zając, Teresa Sembratowicz, Tadeusz Martynkien, Marcin Śmiglak, Katarzyna Komorowska

**Affiliations:** 1 Lukasiewicz Research Network—Institute of Microelectronics and Photonics, al. Lotników 32/46, 02-668 Warsaw, Poland; 2 Department of Optics and Photonics, Faculty of Fundamental Problems of Technology, Wroclaw University of Science and Technology, 27 Wybrzeze Wyspianskiego Str., 50-370 Wroclaw, Poland; 3 Poznan Science and Technology Park, Rubiez Str. 46, 61-612 Poznan, Poland

**Keywords:** ionic liquids, nanoimprint, lithography, grism, grating prism

## Abstract

We present a method of microstructure fabrication on the tip of the optical fiber using a UV soft-imprint process of polymerizable ionic liquid-based optical resist. Ionic liquid with two UV-sensitive vinylbenzyl groups in the structure was diluted in non-hazardous propylene glycol (PG) to obtain liquid material for imprinting. No additional organic solvent was required. The impact of propylene glycol amount and exposure dose on optical and mechanical properties was investigated. The final procedure of the UV imprint on the optical fiber tip was developed, including the mold preparation, setup building, UV exposure and post-laser cure. As the IL-containing vinylbenzyl groups can also be polymerized by the radical rearrangement of double bonds through thermal heating, the influence of the addition of 1–2% BHT polymerization inhibitor was verified. As a result, we present the fabricated diffraction gratings and the optical fiber spectrometer component—grism (grating-prism), which allows obtaining a dispersion spectrum at the output of an optical in line with the optical fiber long axis, as the main component in an optical fiber spectrometer. The process is very simple due to the fact that its optimization already starts in the process of molecule design, which is part of the trend of sustainable technologies. The final material can be designed by the tailoring of the anion and/or cation molecule, which in turn can lead to a more efficient fabrication procedure and additional functionalities of the final structure.

## 1. Introduction

The grating prism can be described as a diffraction grating that is placed on one side of the prism [1]. The main advantage of this type of structure is that the dispersion curves of the prism and the diffraction grating add to each other and can be used in different applications. It is possible to design a structure in which the slope of the dispersion curves of particular GRISM components (grating and prism) for a specific spectral range is canceled [2]. Another interesting attribute is the possibility of in-line propagation of a particular wavelength [1]. Today, gratings and grating-prisms are widely used in astronomical spectroscopy, which, in this case, is called slitless grism spectroscopy. In fact, in the near-infrared camera of the James Webb Space Telescope, silicon grism was used for wavefront sensing purposes [3]. Other applications such as compression of ultra-short pulses [4], chromatic control in 3D multiplane imaging systems [5], improving the spectral domain optical coherence tomography sensitivity [6] or line-by-line beam shaping for more than 600 optical frequency comb lines of a mode-locked Ti:sapphire laser [6] were also proposed. In our contribution, we design and fabricate grism, which is attached to the end-face of the optical fiber. This approach can have several major advantages, such as significant size miniaturization of the device, cost reduction, easy alignment, and minimized instabilities. It could be used to fabricate cheap and small components for spectroscopy [2]. Another interesting application could be coupling light into the waveguide of photonic integrated circuits. With a proper structure design, the bandwidth of a coupler could be broadened [7].

**Figure 1:**
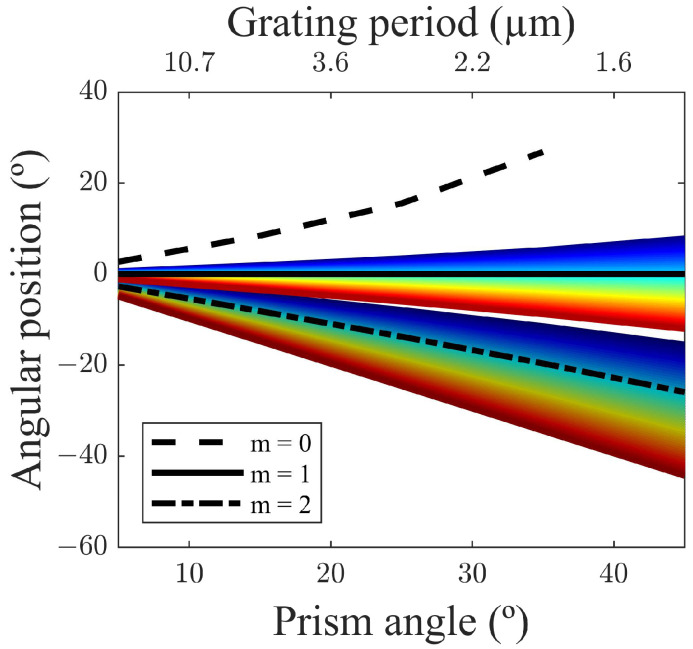
Angular position of m = 0, 1, 2 diffraction order as a function of the solution of Equation (1) for λ = 500 nm (black lines) and wavelengths in the VIS spectrum.

The position of diffraction orders can be calculated from the grating equation [2]:

(1) $$sin\emptyset_{m}=n_{prism}sin\alpha -m\frac{\lambda }{\Lambda }.$$

where ∅m is the angle of diffraction, Λ is the period of the grating, *m* is the diffraction order, α is the angle of the prism, *λ* is the wavelength and $n_{prism}$ is the prism refractive index. To achieve in-line propagation for the 1st diffraction order, it is required to substitute ∅1 = 0°. Then, for a given relation of α with Λ, the angular position of the diffraction orders is calculated for the central wavelength (which is *λ* = 500 nm—black lines) and for a spectral range from 400 to 700 nm, for m = 0, 1 and two diffraction orders. The results are plotted in Figure 1.

As can be seen, the smaller the prism angles, the higher the grating periods required. This period has to be well chosen depending on the optical fiber core size. It can also be seen that the range of angular positions of visible light widens with larger prism angles. Thus, the selection of a larger prism angle is required for efficient spectroscopy application as it increases the resolution of the device. Moreover, the separation between adjacent diffraction orders increases when the grating period decreases.

In order to create a grim structure at the tip of the optical fiber, the material must meet several important conditions—it must be transparent for the working wavelengths, replicate sub-micrometer structures (grating) and, at the same time, allow the formation of a thick wedge-shaped layer (prism).

Keeping in mind the technology cost issues, the requirements of simplicity of the method must be considered, and that should lead to a more sustainable technology in the future. While the optimized material should be simple, the fabrication technology should also be as simple as possible. Nowadays, sustainability is one of the most important features, which is more than required in cases of new technology development and innovation. The presented method includes a solvent-free ionic liquid-based optical material and a simple patterning method.

Ionic liquids (ILs), often called green solvents or “solvent of the future”, are organic salts that are often used instead of traditional solvents. Most of them can be easily prepared in simple and high atom economy processes at mild conditions and recycled, which makes them sustainable materials. There have been many advanced researches, discoveries and patents in the field of ILs in recent years due to their significant features and great applicability, but their commercial exploitation is still limited. Ionic liquids have many unique properties, including low vapor pressure, high thermal stability, low toxicity, high ionic conductivity and almost unlimited tunability, which have already allowed them to find many applications in chemistry, electrochemistry, extraction and separation processes [8,9,10,11,12,13,14]; however, the photonic application of these materials have so far been very limited, and their optical parameters have not been sufficiently explored. Their properties can be tuned either by anion and/or cation modification, making them photodefinable, transparent, colored or flexible. In order to facilitate the patterning of ILs, it has previously been shown that ILs containing allyl groups can be polymerized by means of an electron beam in the process of free radical polymerization or crosslinking [15,16]. Several components made from solidified ILs have been presented, including periodic structures, ring resonators and waveguides [17,18] obtained with e-beam lithography and the focused ion beam (FIB) technique. Ionic liquids are also perfect candidates for creating hybrid systems with nanoparticles (NPs), enhancing their physicochemical properties through intermolecular interactions. ILs have been widely used as a dispersion media and have participated in the synthesis of nanoparticles [19].

The ionic liquids designed for optics and photonics, and to be photodefinable, must have at least three significant features—liquid state with modifiable viscosities (preferably without a solvent), UV-sensitive groups for hardening under irradiation (negative resist) and good optical properties—low optical losses in working range and good thermal stability. In the case of ionic liquids, due to the wide variety of structures of possible molecules, all these requirements can be designed and matched in only one compound instead of the mixtures of various monomers used as traditional nanoimprint materials [20]. During the molecules’ design process, we have to take into account the impact of the number of polymerizable groups, the arrangement in the ionic liquid molecule, and the anion impact on the process and adhesion to the substrate. From polymerizable groups available as chlorides that can be used in the synthesis of quaternary salts, only 4-vinylbenzyl chloride is susceptible to UV polymerization. The number of polymerizable groups has a significant impact on the sensitivity of the resist and the flexibility and durability of the final—solidified—structures. The nature of the anion decides the hydrophilic/hydrophobic nature of the ionic liquid. Halides, such as chlorides or bromides, are highly hydrophilic compounds, which is why they enable easy adhesion to different substrates (glass, polymer or silicon) and elimination of adhesion promotors in the flow process. Additionally, viscosity is also of great importance in imprint resolution because it affects ionic liquid charge transport capacity. It can be modified by changing the cation structure, introducing longer alkyl chains, or switching from aliphatic-based ILs to aromatic-based ILs. Usually, we have to find a compromise between viscosity, density, sensitivity and resolution of the material.

The very preliminary results for IL with one polymerizable group were already shown for a proximity optical lithography process, showing that UV hardening is possible [21]. Ionic liquid can be patterned with a UV imprinting technique, which is considered a cheaper and lower energy alternative than standard optical lithography or electron beam lithography as it relies on the replication of a master mold pattern. The method constitutes one of the most important patterning techniques and has found a widespread application in the fabrication of solar cells [22], optoelectronic and photonic devices [23,24,25]. It has been shown that titania nanoparticles (NPs) prepared in solution with ionic liquid can be directly imprinted by UV exposure using a flexible PDMS mold. As a result, a film structure formed by polymerized, rigid liquid containing stabilized NPs with 2 µm resolution was obtained, showing resistance to cracking and a small shrinkage rate compared to traditional resists [26]. NPs can significantly extend the range of the refractive index values for this material platform.

The process of NIL patterning relies on pressing a mold in the imprint material, which is subsequently cured, whether by temperature (T-NIL) or UV light exposure (UV-NIL). The significant advantages of NIL, including high throughput, low cost and high resolution, have drawn the attention of many researchers, resulting in the development of different mold and resist materials [27,28]. For the latter, materials with various interesting properties were studied in recent years, such as shape memory materials, semicrystalline and liquid polymers or porous materials [29]. The imprinted pattern can form a very fine, wedge-shaped type of structure of the final optical or photonic structures, as well as lenses, waveguides, and diffraction elements, or create special surface shapes in metasurfaces or in free-form optics applications [30].

In this paper, the prism grating made from IL resist on the optical fiber and its spectral characterization are shown. The study is focused on optical IL material with low absorption in the visible and near-infrared range. We show a straightforward method of fabrication of optical diffraction components on the optical fiber tip and the flat surface. We propose a UV imprint of ionic liquids resists containing vinyl groups [31], prepared with propylene glycol without any standard solvent, which makes the process safe and environmentally friendly. No pre- and post-bake of the material is needed. Fabricated structures have a lateral resolution of less than 1 µm. Using the grating equation, we determined the angular positions of the diffraction peaks and compared them with experimentally obtained far-field light distributions. The very good agreement between the analytical and experimental results indicates the high quality of the fabricated structures and the usefulness of IL technology for nanoimprint lithography and allows for the design of more complex devices integrated on the end-face of an optical fiber.

## 2. Results

**Figure 2:**
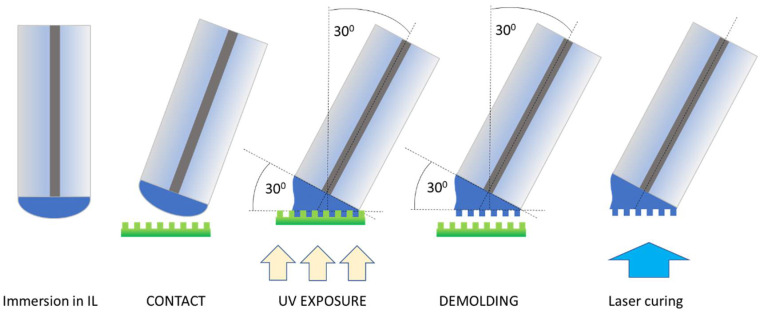
The scheme procedure of a diffraction pattern on a fiber fabrication.

The procedure of grating-prism fabrication on ionic-liquid-based material consists of a few key steps, which are shown in Figure 2. 

### 2.1. Material Immersion

**Figure 3:**
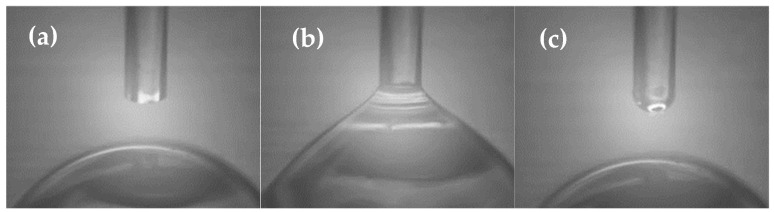
The CCD camera-recorded immersion process: (**a**) the optical fiber end slightly above the droplet of IL, (**b**) the contact with the surface of the ILs droplet, (**c**) small droplet of IL on the end of optical fiber ready for imprinting.

The end of the fiber was immersed in the ionic liquid resist droplet deposited on a smooth flat surface (Figure 3a,b). The process was controlled with the CCD camera to have a very precise amount of the material on the tip. After taking the fiber out (Figure 3c) of the droplet, the flat PDMS was replaced with a patterned PDMS stamp. The same camera allowed for controlling the location of the grating regarding the fiber holder tilt angle. The fiber was brought into contact with the PDMS stamp.

### 2.2. Exposure and Demolding

After that, the structure was illuminated with a UV lamp with a central wavelength λ = 365 nm, and after 10 min of exposure, the substrate was removed.

### 2.3. Optional Post-Laser Cure

When using the side illumination, and due to the fact that for a high prism angle, the layer of IL is much thicker on one side of the grating-prism, an additional and optional exposure was performed after demolding to ensure the full hardening of the material. The 405 nm laser (20 mW) directly from the bottom of the structure was used for this purpose for an additional 2 min.

**Figure 4:**
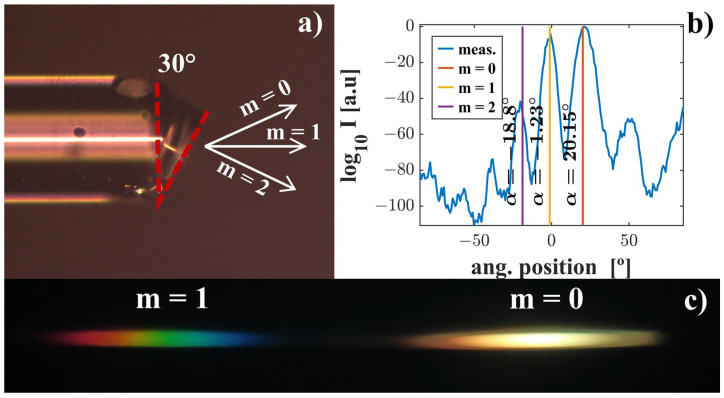
Microscopic side photo of fabricated structure marked with the angle of the prism and direction of diffraction orders (**a**). Far-field diffraction pattern at λ = 516 nm marked with the position of diffraction peaks calculated analytically (**b**). Zero-order and first-order diffraction spectra of supercontinuum source recorded by means of CCD camera (**c**).

In order to achieve in-line propagation for λ = 500 nm, we fabricated a grism at the end of the fiber with a prism angle equal to α = 30° and Λ = 1.8 µm. For these parameters, the angular separation of adjacent diffraction orders is relatively large (m_0_ = 20.25°, m_1_ = 0° and m_2_ = −16.63°). We used a soft mold made of PDMS with a period of 1.8 μm and a relief depth of 400 nm. After several attempts, high-quality periodic structures and the ability to control the prism angle (from 0 to 45°) was achieved. Figure 4a shows a side photo of the fabricated structure marked with the prism angle. To investigate the diffraction efficiencies (of m = 0, 1, and 2 diffraction orders), we used the method described in [32] to measure the light distribution in the far field. The results for λ = 516 µm (normalized with respect to the zero-order diffraction efficiency) are presented in Figure 4b. The positions of the diffraction peaks calculated analytically are indicated in the graph. Figure 4c presents a photo of zero- and first-order diffraction spectra of a supercontinuum source.

## 3. Discussion

The positions of the measured peaks are in very good agreement with the theory, which confirms the high-quality periodic structure fabrication and the ability to control the angle of the prism with high accuracy. Therefore, ionic liquids can be successfully used to produce functional structures on the front of an optical fiber.

Since the component was designed to work in the visible and near-infrared range, the ionic liquid had to fulfill the optical requirements of low absorption and high transmission for this range, and for the process, its physical state should be liquid at room temperature. 

We synthesized and tested different geometries of the cation part, with one and two polymerizing bonds. As a first test, the optical dispersion and absorption of the material layer were measured, as well as the material’s ability to create durable structures under mercury lamp exposure (1000 W) and a UV 365 lamp. For that reason, the thin layers of these materials were prepared and measured by means of optical spectroscopy and ellipsometry.The choice of the cation and anion, as well as the polymerizing group, affects not only the optical and mechanical properties of the material but also the adhesion of the material to the substrate, i.e., the optical fiber.

## 4. Materials and Methods

The material was selected for good adhesion to glass and quartz, good transmission and no tinting, and the ability to work at room temperature. The studied ionic liquids are presented in Table 1.

### 4.1. Synthesis

All used reagents and solvents of high purity were commercially available, purchased from Merck and Iolitec and used as received. NMR spectra were recorded using a Bruker Ascend 400 MHz spectrometer (Billerica, MA, USA) with the utilization of commercially available deuterated solvents.

The chromatographic analyses were carried out using a Metrohm Eco IC system (Metrohm, Herisau, Switzerland) equipped with an 863 Compact IC Autosampler, a 10.0 µL injection loop and a conductometric detector (maintained at room temperature). A self-regenerating Suppressor Module (MSM) (Metrohm, Herisau, Switzerland), regenerated with distilled water and sulfuric acid, was used to separate and determine the anions in ionic liquids. All data were recorded by Metrohm software. An anion chromatograph apparatus was equipped with a Metrosep A Supp 10 ion exchange column (150 × 4.0 mm) coupled with Metrosep A Supp Guard. The packing material was a polystyrene-divinylbenzene copolymer with quaternary ammonium groups. A flow rate of 0.9 mL/min was used. Anion separation was performed with an eluent composed of 30:70, *v/v* proportions of acetonitrile (HPLC purity grade) and an aqueous solution containing 3.2 mM of sodium bicarbonate and 1.0 mM of sodium carbonate (Merck). The eluent was composed of a 30:70 ratio of acetonitrile (HPLC purity grade, Merck), and an aqueous solution containing 0.7 mM of dipicolinic acid and 1.7 mM of nitric acid (Merck). All aqueous solutions were prepared carefully using distilled water (conductivity = 0.05 µS/cm). During cation separation, average pressure in the analytical system was maintained at the level of 3.5 MPa, whereas during anion analysis, the system worked under the pressure of 13.5 MPa. Samples for anion analysis were prepared by dissolving about 15 mg of ionic liquid in eluent solution (3 mL), which was then filtered through a syringe filter. After that, 200 µL of filtrate was taken for analysis into a plastic vial and refilled with 10 mL of eluent. A sample for cation analysis was prepared by the uptake of 200 µL of filtered substance to a vial and then fulfilled with 2.0 mL of acetone (HPLC grade, Merck), 3.0 mL of nitric acid (2.0 mM) and 5.0 mL of proper eluent. Before filtration, cation samples were dissolved in acetone (3 mL) and protonated with nitric acid (2.0 mM).

The purity of all obtained ionic liquids has been determined on the basis of the chromatographic data for cations and anions obtained from ion chromatography analyses. Based on those values, the most accurate purity was calculated according the following formula:

$$\%QA=\frac{Q\times A}{100\%}$$

where *Q* is the quaternary cation purity (%), and *A* is the halide anion purity (%).

Melting point (T_m_) and polymerization temperature (T_max_) were determined by performing DSC experiments with the use of the Mettler Toledo differential scanning calorimeter (Greifensee, Switzerland), cooled with an immersion cooler. The calorimeter was calibrated for temperature and cell constants using high-purity indium (melting temperature: 156.7 °C; specific enthalpy of melting: 28.71 J·g^−1^) and zinc (melting temperature: 419.6 °C; specific enthalpy of melting: −107.5 J·g^−1^). Data were collected at atmospheric pressure. Each sample was analyzed with two heat/cool cycles from 25 to 300 °C at 10 °C·min^−1^. For all experiments, samples in the weight range between 10 and 15 mg were used in aluminum sample pans, sealed with lids with a pin hole. An empty sample pan served as the reference. The temperatures reported for the glass transition, crystallization and melting were established as the onset and peak temperatures, respectively, for the endothermic changes in heat flow. The temperatures reported for T_onset_, T_max_ and T_end_ were established based on exothermic changes in heat flow.

### 4.2. Synthesis of N,N,Nʹ,Nʹ-Tetramethyl-N,Nʹ-bis(4ʹ-Vinylbenzyl)Propane-1,3-Diammonium Dichloride [(N_11_VB)_2_Pr][Cl]

**Figure 5:**
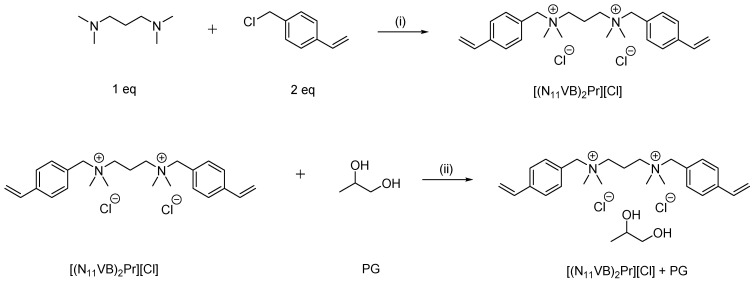
Reaction scheme for the synthesis of ionic liquid and preparation of its mixture with propylene glycol; (**i**) 40 °C, MeCN, 48 h; (**ii**) anhydrous methanol, stirring, evaporation.

*N,N,N′,N′*-Tetramethyl-1,3-propanediamine (3.0 g, 0.023 mol) was dissolved in acetonitrile (40 mL) and heated at 60 °C with simultaneous stirring. Then, 4-vinylbenzyl chloride (1.73 g, 0.05 mol, 7.14 mL) was added dropwise. The mixture was stirred for 48 h, after which the solvent was evaporated under reduced pressure and a yellowish solid was obtained (8.5 g, 85%). The synthesis scheme of studied ionic liquid and preparation of its mixture with propylene glycol is shown in Figure 5.

T_m_ = 240.02 °C.

^1^H NMR (400 MHz, DMSO-*d*_6_) δ 7.62 (s, 8H), 6.81 (dd, *J* = 17.7, 10.9 Hz, 2H), 5.96 (d, *J* = 17.7 Hz, 2H), 5.38 (d, *J* = 11.1 Hz, 2H), 4.75 (s, 4H), 3.51–3.26 (m, 6H).

^13^C NMR (101 MHz, DMSO-*d*_6_) δ 139.32, 136.33, 133.89, 127.99, 126.98, 116.68, 66.77, 60.32, 49.73, 17.37.

Temperatures of polymerization for [(N_11_VB)_2_Pr][Cl] are shown in Table 2. 

### 4.3. Ionic-Liquid-Based Material Preparation

The hardening process and shape stability analysis were carried out on flat glass substrates to facilitate the finding of appropriate doses and enable shape analysis using contact and optical profilometers on larger surfaces as well. As a soft mold, we used a PDMS mold, which was prepared in the following manner: silicon 4″ wafer was covered with 600 nm of positive electron beam resist, exposed and developed. The resist pattern was filled with PDMS material (Sylgard 184, Dow Chemicals) and baked for 3 h at 100 °C. The mold contains optical components such as gratings, lenses and waveguides, and the smallest feature on the master was 0.5 µm.

Since the obtained ionic liquid was solid at room temperature, it was impossible to use it for imprinting; thus, we decided to prepare a mixture based on the principles of deep eutectic solvent chemistry (DES). They are mixtures of hydrogen bond donors and hydrogen bond acceptors. Employing this principle, we decided to choose propylene glycol because, on top of its hydrogen bond donor properties, it is also a low vapor pressure and non-toxic liquid. Furthermore, for the present application, properties such as viscosity close to water viscosity and good optical qualities, such as colorlessness and transparency, were crucial for selection as a hydrogen bond donor. Additionally, quaternary ammonium salts are well-known hydrogen bond acceptors. We prepared several mixtures with different molar ratios, all of which are liquid at room temperature, which means that the hydrogen bonds between ionic liquid and propylene glycol were created.

This ionic liquid solution was prepared in two ways for UV imprinting experiments. In the first one, IL was prepared with the same molarity with PG ((1:1), 6.89:6.89 mmol, 3 g QAS:0.52 g PG). Because of the relatively fast polymerization of this IL by itself at room temperature, the IL was also prepared with butylated hydroxytoluene (BHT, Merck), acting as a polymerization inhibitor. The small amount of BHT ((1:1.5) + 0.1% BHT, 6.89:10.34:6.89 µmol, 3 g QAS:0.78 g PG:1.50 mg BHT) was able to stop the undesirable thermal room temperature induced or white-light-induced polymerization.

### 4.4. UV Dose Setting for ILs Films

**Figure 6:**
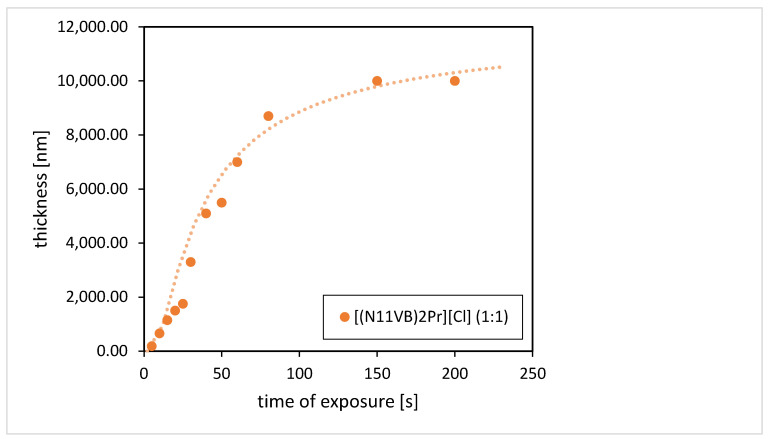
IL-resist contrast curves for photodefinable [(N_11_VB)_2_Pr][Cl] (1:1).

The first step of the experiment was focused on identifying the correct UV dose, which means the dose for which the layer is solidified and resistant to ethanol, to be used as the developing agent. For this purpose, we measured the contrast curves of the IL-resist. Films of ionic liquids were prepared as follows: microscope glass slides were cleaned with ethanol and deionized water and dried on a hotplate. ILs were deposited on the substrate using spin-coating (4000 rpm/s, 40 s). No additional prebake before patterning was performed. The rectangle areas were consecutively irradiated with increasing doses, followed by the development in ethanol for about 15 s. The irradiation was performed using a broadband mercury lamp of 1000 W and lamp intensity of 30 mW/cm^2^ measured at 365 nm (mask aligner MA6 Suss Microtec, Garching bei München, Germany). The dependence of the thickness of the remaining layer after rinsing in ethanol at the time of exposure is shown in Figure 6.

The curve in Figure 6 shows the dose-dependent solidification of the material. For a short amount of irradiation, the material is still partially removable by ethanol. The longer the irradiation time, the more resistant the film. [(N_11_VB)_2_Pr][Cl] starts to polymerize even after short time of exposure of 5 s for an initially 10-μm-thick layer. The final structures of [(N_11_VB)_2_Pr][Cl] are resistant to acetone or ethanol, which indicates that the solidification process involves not only the polymerization of ionic liquid monomers but also the incorporation of glycol propylene molecules in the structure. The optimal exposure dose needed to polymerize [(N_11_VB)_2_Pr][Cl] of 1 μm thickness on the silicon substrate is around 600 mJ/cm^2^. This dose was also recalculated for UV LED lamp exposure at 365 nm (FUTANSI, ShangHai, China), and it took 10 min. After short exposure (shorter than the minimal dose for which the layer was present after development), the layer is polymerized either partially or only on the surface of the thick layer. In such process, the polymerized thin layer will be lifted off after the development process. Such a solidification effect was used before for the fabrication of polymerized suspended structures, and fluorescent containers were shown for the electron beam lithography technique [15,33].

### 4.5. Imprint Patterning Results

**Figure 7:**
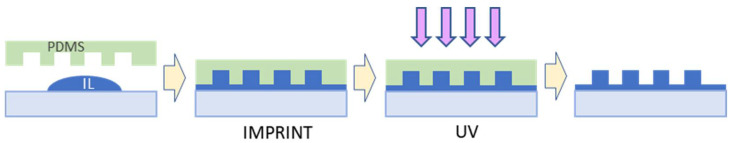
The scheme of the imprint process with PDMS mold.

The imprinted pattern and its durability and optical properties were also tested on microscope slides to be able to measure the dimensions of the pattern and quality of the layer. ILs were spin-coated on a glass or silicon substrate and covered with PDMS mold. Subsequently, the layer was cured with UV and optionally baked on the hotplate (180 °C, 15 min) to obtain the final structure. The scheme of the process is shown in Figure 7. The droplet of the liquid [(N_11_VB)_2_Pr][Cl] with PG in proportions of 1:1.5 (mol/mol) and 0.1% of BHT was covered with PDMS mold and UV cured.

**Figure 8:**
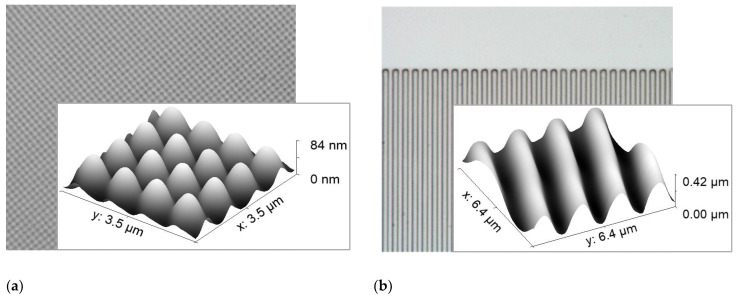
Images of UV imprint of [(N_11_VB)_2_Pr][Cl] + PG. Optical microscopy and AFM image of (**a**) checkerboard pattern, and (**b**) relief diffraction grating 1.7 μm. AFM data was acquired with the use of a Park Systems XE-70 (Suwon, Korea).

The result of UV-NIL is shown in Figure 8. As a control pattern, two structures were chosen: checkerboard pattern and relief grating. The checkerboard relief pattern is reproduced with high resolution (feature size was 0.6 μm) (Figure 8a). For the optical grating, the period was 1.7μm, and the depth of the features measured by optical profilometry was 440 nm (Figure 8b). The smallest obtained period for imprinted grating was 0.6 μm. The UV dose for both imprints was optimized and chosen for 200 s for the 1000 W Mercury lamp, which corresponded to polymerization of the whole layer thickness (on the order of 5–10 μm)—which is much thicker than the depth of the pattern. The vertical shrinkage rate after UV solidification was measured to be 10%.

The imprint of [(N_11_VB)_2_Pr][Cl] was carried out with ethanol or glycol as the pure IL is solid. We noticed that the presence of the propylene glycol changes the mechanical and optical properties of the material, especially the refractive index and the flexibility of the layer. Furthermore, a significant decrease in Young’s modulus measured with AFM (Bruker, Billerica, Massachusetts, USA) after only UV curing indicates higher flexibility of the ionic liquid prepared with propylene glycol compared to the layer obtained from the IL solution with ethanol (shown in Table 3. The pure ethanol-based resist evaporates much faster and hinders the precise control of the process in the case of the structure imprint on optical fibers, so the work was continued on the propylene glycol-based resist.

The additive of propylene glycol can participate in the polymerization process and significantly change the refractive index of the final layer and mechanical properties. It is a known fact that propylene glycol undergoes oxidative degradation under UV irradiation in the presence of oxygen through radical species [34]. These radicals can act as IL polymerization initiators. Moreover, one of the main products of propylene glycol UV-induced decomposition is formaldehyde, which is known to polymerize under UV irradiation [35] and thus can copolymerize with IL in the presented case. It can also be speculated that the PG addition reduces the viscosity of the obtained solution compared to pure IL, which positively affects the mobility of reacting molecules and thus accelerates the reaction itself. The aspects described above are under further investigation.

### 4.6. Optical Properties of [(N_11_VB)_2_Pr][Cl]

**Figure 9:**
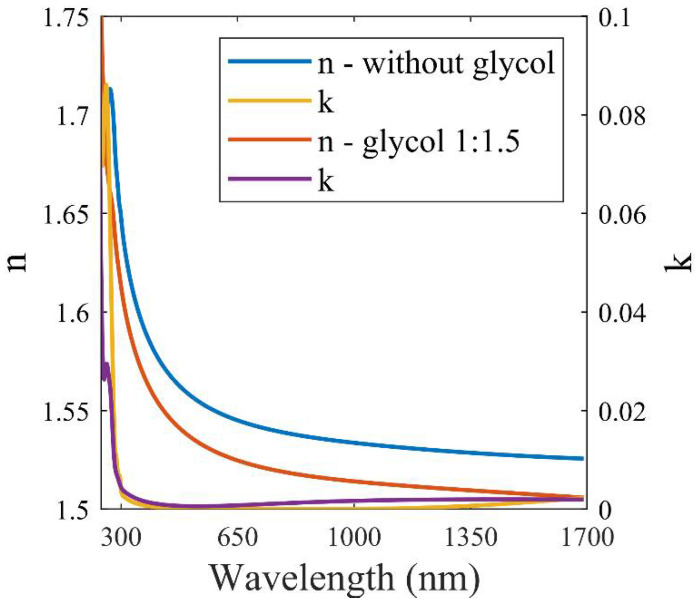
Refractive index (n) and extinction coefficient (k) vs. wavelength of polymerized [(N_11_VB)_2_Pr][Cl] on silicon substrate.

Ellipsometry measurements (J.A. Woollam, Lincoln, Nebraska, USA) of the refractive index were performed for solidified layers of pure IL and for IL with GP. The dependence of the refractive index and extinction coefficient is shown in Figure 9. Table 4 provides the Sellmeier coefficients *A_i_* and *B_i_* that allow us to estimate the refractive index as a function of wavelength (in µm) according to the formula:

$$n{\left(\lambda \right)}^{2}-1=\overset{n=3}{\underset{i=1}{\sum }}\frac{A_{i}*\lambda^{2}}{\lambda^{2}-B_{i}}$$

The presented IL is transparent in the visible range both in a liquid state and after solidification. Furthermore, after polymerization in a volume, it creates a transparent homogenous block of material. Moreover, after complete processing, it is resistant to ethanol or acetone. Layers of polymerized [(N_11_VB)_2_Pr][Cl] are transparent above 300 nm, as shown in Figure 9.

## 5. Conclusions

In this work, we have developed a straightforward technology to fabricate refractive and diffractive structures on a flat surface and on the tip of an optical fiber using a solvent-free optical material ionic liquid and adapted the imprint technique to a new material and optical fiber geometry. An ionic liquid with double vinylbenzyl bonds [(N_11_VB)_2_Pr][Cl] combined with a harmless propylene glycol created a very optically and technologically interesting negative resist as an alternative to the existing optical resists, still often based on more harmful solvents. The material, prior and after UV polymerization, is transparent and has no absorbance in the visible and near-infrared range. Moreover, the final layer flexibility was noticed to depend on the amount of PG in the mixture such that a significant decrease in Young’s modulus is measured when IL is prepared with GP, compared to pure material. IL-based resist facilitates the fabrication process as it does not require any solvent or prebake step. We have shown the scientific application of the presented technique—durable diffraction structures were produced by using a UV imprint on flat surfaces and on the tip of the optical fibers. The optical fiber grism was designed, fabricated and characterized for miniaturized spectroscope application. In future work, we will further develop the material in terms of infrared spectroscopy and material flexibility for applications in more complex geometries and optical fiber structures, as well as on flexible substrates.

## Figures and Tables

**Table 1:** Vinylbenzyl-based ionic liquids used in the study.

Name	Abbreviation	Physical State in Room Temperature	Chemical Structure
N,N,N’,N’-Tetramethyl-N,N’-bis(4′-vinylbenzyl)propane-1,3-diammonium dichloride	[(N_11_VB)_2_Pr][Cl]	solid	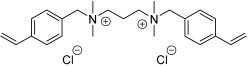
N,N,N’,N’-Tetramethyl-N,N’-bis(4′-vinylbenzyl)propane-1,3-diammonium dichloride + propylene glycol	[(N_11_VB)_2_Pr][Cl] +PG	liquid	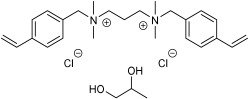

**Table 2:** Temperatures of polymerization for studied ionic liquids.

Compound	T_onset_ (°C)	T_max_ (°C)	T_offset_ (°C)
[(N_11_VB)_2_Pr][Cl]	182.61	201.84	216.17
[(N_11_VB)_2_Pr][Cl] + PG (1:1.5)	121.74	133.77	176.17

(T_onset_—onset polymerization temp.; T_max_—peak polymerization temp.; T_offset_—offset polymerization temp.).

**Table 3:** The comparison of Young’s modulus for UV polymerized layers of IL prepared with glycol and ethanol, measured with AFM in the QNM mode.

	Young Modulus DMT (GPa)	Standard Deviation (GPa)
IL:GP (1:1.5)	20.0	2.5
IL	35.3	3.9

**Table 4:** Sellmeier coefficients.

Sellmeier Coefficients	Without Glycol	Glycol 1:1.5
A1 (μm^2^)	1.25363	1.22158
A2 (μm^2^)	0.08282	0.05359
A3 (μm^2^)	13.83983	0.02213
B1 (μm^2^)	0.01303	0.01426
B2 (μm^2^)	0.05968	0.05767
B3 (μm^2^)	2242.252	7.04113

## Data Availability

Data are contained within the article.
